# Neuropathology of mood disorders: do we see the stigmata of inflammation?

**DOI:** 10.1038/tp.2016.212

**Published:** 2016-11-08

**Authors:** N Mechawar, J Savitz

**Affiliations:** 1 Douglas Mental Health University Institute and Department of Psychiatry, McGill University, Montreal, QC, Canada; 2 Laureate Institute for Brain Research, Tulsa, OK, USA; 3 Faculty of Community Medicine, The University of Tulsa, Tulsa, OK, USA

## Abstract

A proportion of cases with mood disorders have elevated inflammatory markers in the blood that conceivably may result from stress, infection and/or autoimmunity. However, it is not yet clear whether depression is a neuroinflammatory disease. Multiple histopathological and molecular abnormalities have been found postmortem but the etiology of these abnormalities is unknown. Here, we take an immunological perspective of this literature. Increases in activated microglia or perivascular macrophages in suicide victims have been reported in the parenchyma. In contrast, astrocytic markers generally are downregulated in mood disorders. Impairment of astrocytic function likely compromises the reuptake of glutamate potentially leading to excitotoxicity. Inflammatory cytokines and microglia/macrophage-derived quinolinic acid (QA) downregulate the excitatory amino acid transporters responsible for this reuptake, while QA has the additional effect of inhibiting astroglial glutamine synthetase, which converts glutamate to glutamine. Given that oligodendroglia are particularly vulnerable to inflammation, it is noteworthy that reductions in numbers or density of oligodendrocyte cells are one of the most prominent findings in depression. Structural and/or functional changes to GABAergic interneurons also are salient in postmortem brain samples, and may conceivably be related to early inflammatory insults. Although the postmortem data are consistent with a neuroimmune etiology in a subgroup of depressed individuals, we do not argue that all depression-associated abnormalities are reflective of a neuroinflammatory process or even that all immunological activity in the brain is deleterious. Rather, we highlight the pervasive role of immune signaling pathways in brain function and provide an alternative perspective on the current postmortem literature.

## Introduction

The question of whether mood disorders are neuropathological conditions has been the subject of considerable debate.^1, 2, 3^ In the case of major depressive disorder (MDD) and bipolar disorder (BD), there is evidence for subtle histopathological changes observed postmortem that plausibly may affect excitatory and inhibitory circuits involving the prefrontal cortex (PFC) and limbic regions. The most salient of these abnormalities are reductions in the size and/or density of GABAergic neurons and reductions in glial cell densities that are concurrent with altered gene expression. These data have been reviewed in detail elsewhere^1^ and are not comprehensively covered here. Instead we focus on one potential cause of the histopathological changes associated with mood disorders—inflammation. That is, we highlight those postmortem cellular and molecular abnormalities that may be directly or indirectly related to immune dysregulation. This is not intended to be a systematic review of the literature. Nor do we argue that all changes observed postmortem in mood disorders are related to inflammation. Rather we provide an alternative perspective on a substantial and diverse body of work.

## Evidence for immune dysregulation in mood disorders

A large number of studies have reported increased levels of inflammatory proteins such as tumor necrosis factor (TNF), interleukin 6 (IL-6) and C-reactive protein (CRP) in the serum or plasma of depressed individuals with MDD and BD, and these results have been confirmed in meta-analyses.^4, 5, 6, 7, 8^ Concentrations of pro-inflammatory cytokines are also reportedly elevated during mania or hypomania.^9, 10^ Although most studies have focused on cytokine proteins, additional support for the role of inflammation in mood disorders is derived from gene expression studies of peripheral blood mononuclear cells, which have demonstrated the existence of increased messenger RNA (mRNA) expression of pro-inflammatory mediators in patients with mood disorders.^11, 12, 13, 14^ Notably, Padmos *et al.*^11^ performed whole-genome expression profiling on microarrays using purified cluster of differentiation 14 (CD14+) monocytes and reported elevated mRNAs of inflammatory (for example, TNF, IL-1, IL-6, TNF-alpha-induced protein 3), trafficking, survival and mitogen-activated protein kinase pathway genes in BD subjects in various illness phases, and in the affected offspring of other BD subjects. Similarly, the expression of several genes previously implicated in neurological and inflammatory disorders, including TNF, were found to be upregulated in the peripheral blood mononuclear cells of a combined sample of depressed MDD and BD participants.^14^ Further, Pandey *et al.*^13^ found that the mRNA expression of IL-1, IL-6 and TNF, as well as their receptors, IL-1R1, IL-1RA and TNFR1 was significantly greater in the lymphocytes from BD patients than those of healthy controls. These data demonstrate the potentially important pathological role of membrane-bound cytokine receptors, which mediate the functional and biological effects of cytokines. In a follow-up study by the same group, similar increases in the mRNA expression of cytokines and their membrane-bound receptors were found in hospitalized MDD patients.^15^ Soluble cytokine receptors may also be relevant to the pathophysiology of depression. Elevations in the protein concentrations of soluble IL-1R (sIL-1R), sTNFR1, sTNFR2 and sIL-6R also have been reported in patients with mood disorders.^16, 17, 18^

It is not yet clear whether the association between mood disorders and inflammation is causal. Regarding prospective studies, elevated CRP levels have been shown to predict the subsequent development of BD^19^ as has been demonstrated several times for CRP or IL-6 in the case of MDD and psychosis.^20, 21, 22^ Second, it has been well established that treatment of hepatitis C or melanoma patients with interferon alpha (IFN-α) or interleukin 2 (IL-2) induces a depressive episode in about 40% of patients.^23, 24, 25^ Importantly, there is a temporal disjunction between the ‘psychological' and ‘physical' manifestations of this immune-stimulating treatment, with the neurovegetative symptoms appearing within 1 week, whereas the mood and cognitive symptoms peak 8–12 weeks post initiation of treatment.^26, 27^ Moreover, it is the mood and cognitive symptoms rather than the neurovegetative symptoms that are responsive to antidepressant treatment.^26, 27^ Similarly, the typhoid vaccine and low-dose endotoxin, which has been shown to activate microglia *in vivo*,^28^ induce transient, mild depressive symptoms in healthy controls.^29, 30, 31^ These data prompted investigations into the efficacy of anti-inflammatory medications for the treatment of bipolar depression. Results have been mixed^32^ although low-dose aspirin, minocycline, infliximab and n-acetyl cysteine show some therapeutic promise.^33, 34, 35^ Finally, positron emission tomography studies have found evidence for microglial activation in the PFC and anterior cingulate cortex (ACC) of patients with MDD as well as the hippocampus in patients with BD as indexed by an increase in the distribution volume of the ligand for the translocator protein ligand, TPSO, which is expressed by microglia.^36, 37^

Inflammatory mediators likely affect neuronal function and neurotransmission by an array of different mechanisms. For instance, pro-inflammatory cytokines such as interferon gamma (IFN-γ), IL-1 and TNF can reduce the availability of monoamines (serotonin, dopamine and norepinephrine) by upregulating synaptic reuptake transporters, as well as reducing monoamine synthesis by decreasing the availability of tetrahydrobiopterin (BH4), a co-factor for the enzyme tyrosine hydroxylase (see ref. 38 for a comprehensive review). At the circuit level, these alterations in neurotransmission lead to functional changes in components of the visceromotor network including the ventromedial PFC, insula and hippocampus,^30, 31, 39^ as well as hypoactivity of a ‘reward network' centered on the ventral striatum, thus providing a clear link with the behavioral trait of anhedonia.^29, 40, 41^ Nevertheless, postmortem studies have generally pointed to abnormalities in glutamatergic and/or GABAergic circuits in BD, and thus immune-mediated modulation of the balance between excitatory and inhibitory neurotransmission will be the primary focus here.

**Figure 1:**
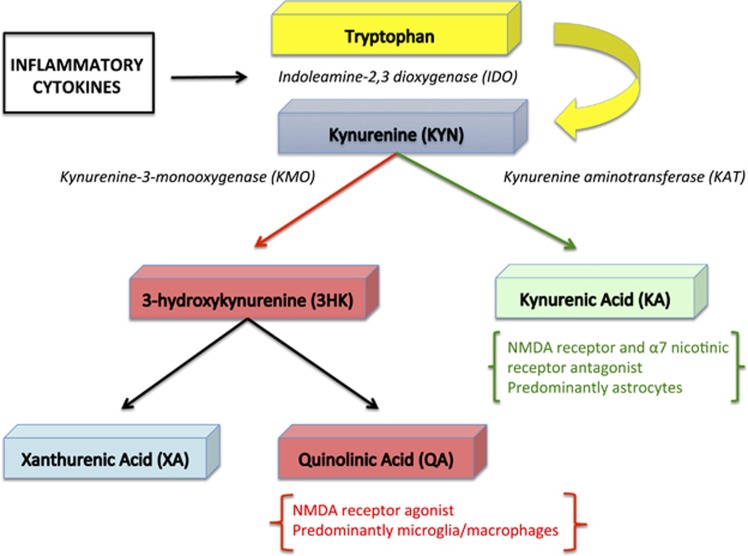
Main branches of the kynurenine pathway. The enzyme indoleamine 2,3 deoxygenase (IDO), which converts tryptophan to kynurenine is upregulated by pro-inflammatory cytokines. Each box represents a metabolite resulting from the oxidation of tryptophan. The black italicized text shows the enzymes that catalyze select steps in the metabolic pathway. NMDA, *N*-methyl-d-aspartate.

As will be discussed below, activated microglia release glutamate^42, 43, 44, 45, 46, 47^ and pro-inflammatory cytokines further elevate the risk for excitotoxicity by impairing astrocyte-mediated glutamate recycling.^48, 49^ However, inflammatory mediators also may affect glutamatergic signaling indirectly by, for instance, altering the production of neuroactive metabolites of the kynurenine pathway (Figure 1). Specifically, pro-inflammatory cytokines upregulate the enzyme indoleamine 2,3 deoxygenase, increasing the production of kynurenine from tryptophan. Kynurenine in turn is metabolized along two principal branches to form kynurenic acid (KynA) or alternatively, the potentially neurotoxic metabolites, 3HK (3-hydroxykynurenine) and quinolinic acid (QA).^50^ KynA is a pleiotrophic metabolite that is produced by astrocytes and among other roles, acts as a preferential antagonist at the glycine co-agonist site of the *N*-methyl-d-aspartate (NMDA) receptor.^51^ In contrast, QA is produced by microglia and is neurotoxic at elevated concentrations partly via its agonistic effect on the NMDA receptor.^52^ Although QA is usually found in low nanomolar concentrations in the human brain and cerebrospinal fluid, a significant increase in QA levels to micromolar concentrations is observed in patients with inflammatory neurological diseases.^53^ In addition to its effect on the NMDA receptor, QA has been shown to increase the production of reactive oxygen and nitrogen species,^54^ inhibit the reuptake of glutamate into synaptic vesicles,^55^ promote the formation of hyperphosphorylated tau proteins^56^ and upregulate transcription of pro-inflammatory chemokines and cytokines in astrocytes.^57^

Decreases in KynA and increases in QA in the cerebrospinal fluid of predominantly depressed subjects have been found up to 2 years after a suicide attempt,^58^ and we previously reported a reduction in the ratio of KynA to 3HK and KynA to QA in the serum of patients with both MDD and BD.^59, 60^ In addition, the ratio of 3HK to KynA and/or QA to KynA in the serum was inversely correlated with hippocampal volume in both MDD and BD participants, as well as depressed football players with concussion, raising the possibility that neuroactive kynurenine metabolites may affect brain structure in the context of depression.^59, 60, 61^

## Etiology of the immune dysregulation in mood disorders

Although the evidence for immune dysregulation in a subgroup of individuals with mood disorders is robust, the upstream cause of this immune dysregulation is less clear. Below, we discuss three potential, non-mutually exclusive causal factors, stress, pathogens and autoimmunity.

### Stress

Stress hormones (for example, epinephrine, norepinephrine and cortisol) and ‘hard-wired' sympathetic nervous system innervation of lymphoid organs exert pleiotrophic effects on the immune system via their receptors on immune cells. This stress response causes (potentially chronic) ‘low-grade' inflammation characterized by increases in pro-inflammatory mediators such as TNF, IL-6 and IFN-γ, as well as impairment of the adaptive immune system leading to increased vulnerability to viral disease, deficient response to vaccination and poorer prognosis for diseases such as cancer.^62^ In complementary studies, Cole and colleagues have demonstrated that social isolation and loneliness is associated with a consistent pattern of gene expression in peripheral blood leukocytes, that is, an increase in inflammatory markers involved in wound healing and bacterial immunity together with a downregulation of transcripts involved in Type I interferon antiviral responses and IgG antibody production.^63, 64, 65^ In the brain, this immunological dysregulation may lead to increased activity of the amygdala, deficient hippocampal neuroplasticity, and more generally, morphological and/or atrophic changes to pyramidal neurons.^66^

### Pathogens

The impaired adaptive immunity associated with chronic stress may lead to increased susceptibility to the negative sequelae of pathogens, especially viruses. Herpesvirus infections are a salient example. The majority of the population of the United States (US) is seropositive for Epstein–Barr virus and herpes simplex 1 virus,^67^ whereas more than half of the US population is seropositive for cytomegalovirus.^68^ Generally, these viruses remain latent after initial infection but they may undergo reactivation during periods of both physical and psychological stress without producing significant clinical disease. Reactivation is usually accompanied by a noticeable increase in specific antibody titer to the virus, even in the absence of detectable virus. For instance, IgG antibody titers to cytomegalovirus, Epstein–Barr virus and herpes simplex 1 virus were found to be significantly elevated in medical students during exams compared with when they returned after summer vacation.^69^ Several studies have reported higher IgG cytomegalovirus antibody titers in depressed subjects relative to controls^70, 71, 72^ and more recently, the Detroit Neighborhood Health Study showed that individuals with cytomegalovirus antibodies in the top quartile were four times more likely to be depressed than those individuals in the bottom three quartiles of the population.^73^

Another pathogen that may have a role in the pathophysiology of mood disorders is the protozoan, *Toxoplasma gondii*.^74^ A meta-analysis of 23 studies found that serological evidence of infection with *T. gondii* was associated with a 2.73-fold increased risk of schizophrenia^75^ and several recent studies have reported associations between *T. gondii* seropositivity and depression, mania and suicidal behavior more generally.^76, 77, 78, 79^ Like the herpesviruses, *T. gondii* is neurotrophic and additionally encodes proteins with homology to tyrosine hydroxylase and the D2 receptor, raising the possibility that it may modulate dopaminergic neurotransmission.^80^ Further, *T. gondii*-infected mice display increased production of neuroactive kynurenine pathway metabolites in the brain, suggesting another possible mechanistic connection between *T. gondii* and the pathophysiology of mood disorders.^81^

### Autoimmunity

Certain microbial infections may increase the risk of developing autoimmune disease via molecular mimicry and/or bystander activation and stimulation of pattern recognition receptors.^82^ There are two predominant sources of evidence for autoimmune illness in depression and psychosis. First, there is a greater prevalence of various autoimmune disorders in patients with mood disorders than that of the general population.^83, 84, 85, 86, 87^ Further, there is persuasive epidemiological evidence to suggest that autoimmune disease is a risk factor for the development of *de novo* mood disorders. For instance, using the Danish Psychiatric Central Register, which included data on 90 000 inpatient admissions for depression, Benros *et al.*^88^ found that a history of any prior autoimmune disease increased the risk of a subsequent diagnosis with a mood disorder by 45%, a history of hospitalization for infection increased the risk of later mood disorders by 62% and the two factors interacted in synergy to increase the risk of subsequent mood disorders by 235%.

Second, there is evidence to suggest that a subset of patients with mood disorders have elevated levels of circulating autoantibodies. For instance, thyroperoxidase antibodies are significantly more common in patients with MDD and BD even after accounting for lithium exposure,^89, 90^ a finding that may be related to a shared genetic vulnerability to BD and autoimmune thyroiditis.^84^ In another one of many examples, Ching *et al.*^91^ reported that 2 out of 20 patients with a diagnosis of MDD had raised levels of autoantibodies to glutamic acid decarboxylase 65 (GAD_65_) and Ro_52_ in both the serum and cerebrospinal fluid, with one of the patients meeting criteria for stiff-person syndrome. Whether these antibodies have a causal role in the development of the illness or are a consequence of an independent pathological process is still unknown. However, at least some cases of psychosis and/or depression appear to be caused by antibodies that have functional effects on neurotransmission. Dalmau and colleagues first reported the existence of autoantibodies, which cause cross-linking and internalization of the NMDA receptor in limbic encephalitis patients with a variety of psychiatric symptoms including auditory and visual hallucinations, delusions, depression and mania.^92, 93^ Further, ~4% of the cases in the Kayser *et al.*^93^ series presented with isolated psychiatric symptoms. Subsequently, anti-NMDA receptor antibodies also have been reported to be present at an increased frequency in patients initially diagnosed with MDD (3%)^94^ and post-partum psychosis (2%).^95^ On the basis of these and other studies, it has become increasingly recognized that a small minority of patients diagnosed with psychosis or depression may in fact have a variant of autoimmune encephalitis. In a recent editorial, Lennox *et al.*^96^ wrote that ‘Antibody screening in young people presenting with psychosis, seizures and cognitive disturbance is now part of routine clinical practice in neurological and intensive care settings [in the United Kingdom]'.

## Inflammation-related histopathological abnormalities

### Postmortem brain studies: methodological considerations

Among the different approaches used to investigate psychiatric illnesses, postmortem brain research offers a unique window through which underlying cellular and molecular alterations can be observed and described with the highest resolution and sensitivity. Moreover, postmortem studies can provide invaluable information on cerebral cells and networks that are only found in humans. This is particularly crucial when it comes to mental illnesses unique to humans such as BD. For instance, our view of human astrocytes has changed considerably in the past few years since Nedergaard and colleagues elegantly highlighted that both the size and complexity of cortical astrocytes are disproportionately greater in humans than in rodents.^97, 98^ Furthermore, the same team found that the diversity of these cells is more important in the human neocortex, with astrocytic subtypes displaying projections spanning several cortical layers or columns. The great diversity of human astrocytes was subsequently confirmed and extended to other brain regions, with the discovery of novel subtypes in the hippocampus and, more recently, in subcortical regions such as the thalamus and caudate nucleus.^99, 100^ The notion that human astrocytes may also display different functional properties than in animal models further highlights the importance of clinical and postmortem studies to better understand the biological roots of mental illnesses.

Despite these advantages, postmortem studies also present some limitations, and the results should always be interpreted with consideration for factors such as: (i) the quality of sample preservation, which is generally assessed by measuring tissue pH and RNA quality (RNA Integrity Number); (ii) the necessity of adequately matched controls (age, death with or without agonal period, postmortem delay, tissue characteristics and so on); and (iii) the amount of information (for example, clinical, psychosocial and so on) available for each donor, which can be considerably increased by psychological autopsies. Even when generated in optimal conditions, postmortem data showing significant differences between cases and controls should be viewed as a ‘snapshot' of cellular and molecular events unfolding in brain tissues before death, without precluding the possibility that the observations may reflect cumulative changes having occurred during the course of the illness. Consideration should also be given to possible postmortem changes to brain tissues between death and their preservation by freezing or fixation, although a careful matching of samples should eliminate this concern.

### Pro-inflammatory molecules and pathways

Despite the fact that several studies have examined peripheral (plasma and cerebrospinal fluid) inflammatory molecules and mediators in patients with mood disorders, little is currently known about the expression of inflammatory markers in the brain of such individuals. The few postmortem investigations that have addressed this question have all examined cortical areas and are, unfortunately, largely inconsistent. Using real-time polymerase chain reaction to compare the expression of TNF, IL-1, IL-4, IL-5, IL-6 and IL-13 in the orbitofrontal cortex of suicides vs non-psychiatric controls, Tonelli *et al.*^101^ reported a significant increase in the expression of IL-4 in women and of IL-13 in men. As highlighted by the authors, this study had important limitations, such as a lack of diagnosis for the majority of suicides, the absence of toxicological data, and the fact that groups were not matched by age.^101^ The roles of IL-4 and IL-13 in the brain, similar cytokines released by T-helper 2 cells in allergic inflammation, remain mostly to be determined (but see ref. 102). Interestingly, there is *in vitro* evidence showing that in rat cells, IL-13 can lead to the death of activated microglial cells by enhancing the production of cyclo-oxygenase-2.^103^ Thus, increased IL-13 expression could represent a mechanism through which microglial activation is kept in check.

In their investigation of teenage suicides (various diagnoses), Pandey *et al.*^104^ found that both mRNA and protein levels of pro-inflammatory cytokines TNF, IL-1 and IL-6 were significantly increased in prefrontal cortex (Brodmann's Area 10; BA10) relative to matched controls. These findings are consistent with the results of a later study by this group on the expression of the Toll-like receptors (TLR) found on macrophages and microglia.^105^ They showed that the expression (mRNA and protein) of TLR3 and TLR4 was significantly upregulated in the dorsolateral PFC of depressed suicides vs non-depressed suicides. TLR3 and TLR4 mRNA, but not protein, was similarly increased in depressed non-suicides, suggesting that suicide, independent of diagnosis, is associated with a stronger dysregulation in the expression of these receptors.^105^

Focusing on TNF protein expression in prefrontal cortical samples (BA24 and BA46) from 10 adult MDD patients (mostly suicides) and matched controls, Dean *et al.*^106^ also detected a highly significant depression-associated increase in protein levels of transmembrane—but not soluble—TNF that was restricted to BA46. A more extensive follow-up study by these authors showed a greater than three-fold increase in transmembrane TNF expression in ACC (BA24) samples from BD patients, as well as a 51 and 67% decrease in TNFR2 expression in BA46 samples from MDD and BD subjects, respectively.^107^ These results suggest illness-specific regional disruptions in TNF signaling. In contrast, Rao *et al.*^108^ did not detect any difference in TNF expression in the frontal cortical samples from BD patients relative to controls. They did however, report significantly increased mRNA and protein levels for IL-1 and IL-1R, as well as of astrocytic (glial fibrillary acidic protein; GFAP) and microglial (CD11b) markers in the same tissues.^108^ Using microarrays, a more high-throughput approach, some investigators have reported a widespread upregulation of both pro- and anti-inflammatory cytokines in the dorsolateral PFC of MDD patients,^109^ whereas others have been unable to implicate cerebral inflammation in any of brain regions examined in individuals with BD.^110^

The inconsistencies in the literature presented above could be attributable to differences in experimental approaches or to the diversity of postmortem brain samples. As mentioned above, however, this diversity could be further enhanced by the clinical heterogeneity of patients suffering from mental illness. Molecular differences associated with such heterogeneity were recently highlighted in a postmortem study aimed at examining genes involved in stress and inflammation.^111^ Fillman *et al.*^111^ performed postmortem gene expression analyses with Stanley Array Cohort frontal/prefrontal cortex samples (34 BD, 35 schizophrenia and 35 control). Using eight markers of inflammation (Serpin Family A Member 3, IL-1, IL-1R1, IL-6, IL-8, IL-18, TNF and Prostaglandin-Endoperoxide Synthase 2), the authors were able to cluster the samples into a high inflammation subgroup consisting of 16 samples with schizophrenia (46%), 10 samples with BD (29%) and 9 controls (26%), and a low inflammation subgroup consisting of 19 samples with BD, 20 samples with schizophrenia and 25 controls. The samples also were clustered into high and low stress groups on the basis of the expression of several glucocorticoid signaling-related markers. The stress and inflammation markers were further combined to yield a high stress/inflammation subgroup and a low stress/inflammation subgroup with the former consisting of 46% of the schizophrenia group, 32% of the BD group and 18% of the controls. In addition, a microarray analysis demonstrated that 57 genes were differentially expressed between the high inflammation/stress subgroup of psychiatric samples and the low inflammation/stress subgroup of controls. A pathway analysis of these 57 genes identified a network of differentially expressed genes involving immune, growth factors, inhibitory signaling and cell death factors.^111^ Thus, a robust immune activation in the brain does seem to be occurring in subgroups of patients with BD and schizophrenia. More recently, Clark *et al.*^112^ compared the postmortem mRNA expression of various cytokines in ventrolateral prefrontal cortical samples from patients with depression not otherwise specified to matched controls. Unexpectedly, this study showed that the expression of TNF, IFN-γ, IL-13, IL-33, IL-12 and of Chemokine (C-C Motif) Ligand 2 (CCL-2), was significantly decreased in the samples from depressed patients. Furthermore, these findings were correlated with evidence of a compromised kynurenine pathway in the same samples. Taken together, these studies suggest that the important variations in expression levels of inflammation-related molecules and pathways measured in postmortem brain samples potentially reflect the clinical heterogeneity of samples analyzed, both between and within diagnoses.

### Microglia

**Figure 2:**
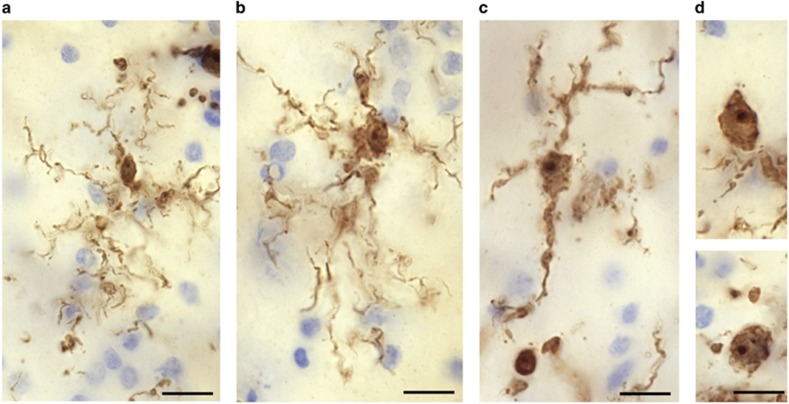
(Torres-Platas *et al.*^116^): Four main IBA1-IR microglial phenotypes are observed in human dorsal anterior cingulate cortex (ACC). Representative examples are illustrated here for the white matter. (**a**) Ramified microglial cell body and highly ramified processes. (**b**) Primed microglia display a wider cell body compared with the ramified phenotype. (**c**) Reactive microglia present an ameboid-shaped rounder cell body with a few ramified processes, whereas (**d**) ameboid microglia display a characteristic ameboid-shaped cell body extending one or two unramified processes (top panel) or are completely devoid of processes (bottom panel). Scale bars, 10 μm. IR, immunoreactive.

Microglia are the resident immune cells of the brain (Figure 2). In their ‘resting' state, these macrophages display a ramified phenotype and are actively implicated in neuronal plasticity.^113^ Upon immune activation, microglia release pro-inflammatory cytokines as well as other factors, such as glutamate, chemokines and growth factors, and can undergo graded morphological changes leading up to a highly motile ameboid phenotype.^114, 115^ Other types of macrophages can be found in the brain, such as infiltrated monocytes and perivascular macrophages, both of which are highly responsive to alterations of the blood–brain barrier integrity in pathological conditions. The best way to distinguish these different cell populations in postmortem brain samples remains the observation of their fine morphological properties and spatial distribution in immunostained tissues.^116^ With this approach, it is particularly easy to distinguish microglia from perivascular macrophages, as the former occupy the parenchyma in non-overlapping domains, whereas the latter are clearly associated with blood vessels.^114, 115^ Although many macrophage-specific markers have been identified, it is currently impossible to differentiate macrophage cell populations based on the expression of these markers alone. The scavenger receptor CD163 may represent an exception, as it is more highly expressed by perivascular macrophages than microglia.^117^

Few studies have examined microglia/macrophages in postmortem samples from individuals having suffered from mood disorders. Steiner *et al.*^118^ provided the first evidence suggesting increased microglial activation in psychiatric illnesses. These authors examined the immunohistochemical distribution of human leukocyte antigen-DR, a major histocompatibility complex (MHC class) II cell surface receptor specifically expressed by macrophages, in samples of dorsolateral prefrontal cortex, ACC and mediodorsal thalamus from individuals having died with MDD, BD and schizophrenia as well as matched non-psychiatric controls.^118^ They observed increased densities of human leukocyte antigen-DR-immunoreactive (-IR) microglial cells in all the three brain regions. Interestingly, this observation concerned samples from individuals having died from suicide, irrespective of diagnosis.^118^ The same group subsequently published another postmortem study showing increased microglial quinolinic acid-IR within ACC subregions of severely depressed individuals compared with matched controls.^119^ More recently, support to the notion of depression-associated microglial/immune activation in the brain was provided by independent postmortem investigations focused on other microglial markers. On the basis of previously established morphometric criteria,^116^ the first of these studies assessed through stereology the relative abundance of microglial phenotypes immunostained for the macrophage-specific calcium-binding protein IBA1 in the dorsal ACC white matter of depressed suicides and matched sudden-death controls.^120^ Although this comparison suggested a relative increase of primed microglia in depressed suicides, the most striking observation was that samples from depressed suicides displayed significant more blood vessels surrounded by a high density of IBA-IR macrophages than matched controls.^120^ This may reflect increased recruitment of circulating bone marrow-derived monocytes in depressed suicides, a phenomenon that has been associated in mice with anxiety-like behavior in response to repeated social defeat stress.^121^ The distinction between resident microglia cells and monocyte-derived macrophages is important because the latter have been more closely linked with neurotoxicity, for instance producing 32 times more QA than resident microglia.^122^

The postmortem study conducted concomitantly by Schnieder and colleagues suggested that the accumulation of macrophages in the perivascular space is not specific to the ACC. Indeed, their stereological investigation of IBA1 and of CD68-IR cells in the prefrontal white matter also showed an increase in perivascular macrophages in suicides relative to controls.^123^ In this study, the group of cases was composed of individuals who had suffered from affective disorders or from schizophrenia, and the results were significant only in the subgroup of patients who died by suicide. It remains to be explored whether this phenomenon occurs solely in the white matter or whether the blood vessels in neocortical gray matter are similarly affected. Moreover, it will be important to determine whether the perivascular space is affected in this manner only in regions associated with mood disorders and suicide, such as the ACC and PFC, or whether this is a more global phenomenon in the brain.

Glutamate released by activated microglia may induce excitotoxicity and contribute to neuronal damage and/or dysfunction. A recent proton magnetic resonance spectroscopy study reported elevations in glutamate in the basal ganglia and dorsal ACC in patients receiving treatment with IFN-α, and higher levels of glutamate correlated with increases in depressive symptoms during the course of treatment.^124^ Hashimoto *et al.*^125^ reported increased levels of glutamate in the frontal cortex of BD patients and several studies identified increases in the glutamine+glutamate (GLX) signal in multiple brain regions in BD patients.^126, 127^ The GLX signal is constituted predominantly by intracellular glutamate and glutamine and is thought to reflect the total glutamatergic pool available for neurotransmission in the form of glutamate or glutamine.^126^ The smaller number of studies that distinguished between glutamate and glutamine, generally suggested elevations in glutamate in patients with BD.^127^ Thus it is unclear how best to interpret the increase in GLX, but the data are at least consistent with glutamate-induced neuronal hyperactivation.^128^

### Astrocytes

**Figure 3:**
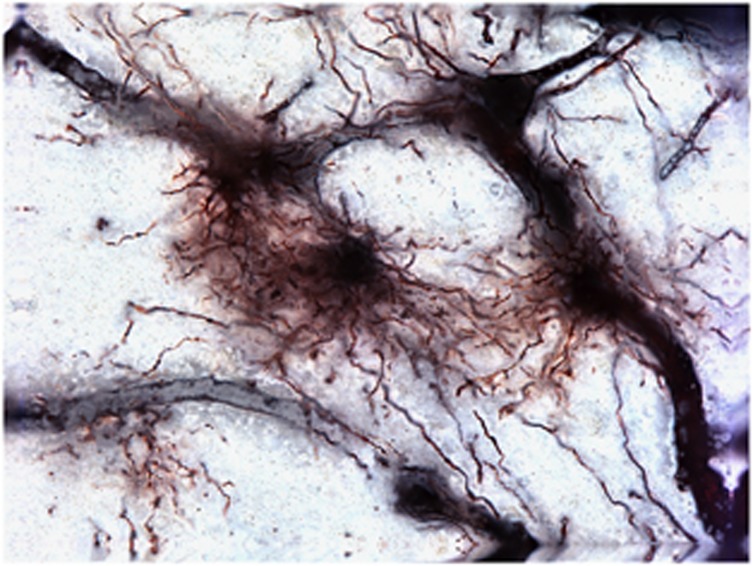
Golgi-stained astrocytes in the human dorsal anterior cingulate cortex (dACC). These cells extend tortuous varicose and thorny processes radiating in all the directions and often are observed to contact adjacent blood vessels, which are also silver-impregnated.

Astrocytes (Figure 3) have attracted much attention in the search for etiological factors in depression.^129^ Consistent with the changes described above concerning macrophages in ACC white matter, morphometric data generated from the analysis of reconstructed Golgi-stained cells indicated that fibrous astrocytes in this cortical compartment displayed a hypertrophic phenotype in depressed suicides (MDD and BD) relative to controls.^130^ This observation may be indicative of mild astrogliosis in response to local low-grade neuroinflammation. In this regard, it is puzzling that postmortem studies examining the expression (gene and protein) of GFAP, an astrocyte-specific intermediate filament known to be upregulated with inflammation, have consistently found it to be significantly decreased in depressed cases vs matched controls.^99, 131, 132^ Initially described in prefrontal cortical areas, this downregulation was also recently reported in subcortical regions implicated in depression, but not in other unrelated cortical areas (for example, primary and visual motor cortex), suggesting that specific networks of astrocytes are affected in mood disorders, and that downregulation of GFAP is not a brain-wide phenomenon.^99^

Beyond GFAP, studies have found that other astrocyte-specific genes such as the tropomyosin-related kinase B receptor (TrkB.1) isoform^133^ and connexins 43 and 30 (refs 134, 135) are significantly downregulated in the orbitofrontal and dorsolateral PFC of suicide completers, respectively. The observation of widespread astrocytic gene downregulation could conceivably reflect a loss of astrocytes. This would be consistent with decreases in densities of GFAP-IR cells reported in prefrontal cortical samples from depressed patients.^131^ Alternatively, these changes could occur without cell loss and instead be owing to stress-induced structural changes in astrocytes.^136^ Although the underlying mechanisms remain to be elucidated, either a loss or a significant atrophy of astrocytes within fronto-limbic brain regions undoubtedly signifies that communication is altered within these networks in mood disorders and suicide. This likely affects the immune functions of these glial cells, and certainly compromises their essential role in the glutamatergic tripartite synapse.

Glutamate reuptake is critical for regulating glutamate concentrations in the synaptic cleft and maintaining normal synaptic activity, and thus an impairment in glutamate transport may result in excessive or dysregulated glutamate receptor signaling. Under physiological conditions, astrocytes prevent excitotoxicity by maintaining extracellular glutamate in the micromolar range via the high-affinity glutamate transporters, excitatory amino acid transporter (EAAT) 1 and EAAT2.^137^ However, this balance may be disrupted by oxidative stress or inflammation leading to necrosis and/or apoptosis through excessive stimulation of glutamate receptors.^49^ Several researchers^42, 43, 44, 45, 46, 47^ have shown that activated microglial cells release glutamate via the system xc− cystine/glutamate transporter (Xc^−^) that exchanges cysteine for glutamate, and that this process may be accentuated by inflammation-induced downregulation of the astrocytic EAAT1 and/or reversed function (that is, glutamate release) of EAAT2, potentially leading to excitotoxicity.^48, 49^

A microarray analysis of samples of the ACC (BA24) and the dorsolateral PFC (BA9 and BA46) from people with MDD showed reduced expression of the genes coding for the high-affinity glutamate transporters glial excitatory amino acid EAAT1 and EAAT2 along with decreased expression of glutamine synthetase, the enzyme that converts glutamate to glutamine.^138^ These results were subsequently extended to the locus coeruleus^139^ and hippocampus.^140^ It is not immediately clear from these studies whether the decrease in glutamate transporter expression was driven by a reduction in glial cell number. However, at least in the case of the locus coeruleus, the depression-associated decrease in EAAT1 and EAAT2 expression was found to be present when expression was normalized to the number of astrocyte cells (but not oligodendrocytes), suggesting an astrocyte-specific dysfunction.^141^

Regarding BD, the picture appears to be more complicated. Both mRNA and protein levels of EAAT1 were reported to be increased, whereas EAAT2 mRNA and protein levels were decreased relative to controls in samples from the frontal cortex (BA10).^142^ As ∼90% of glutamate is removed from the synapse by EAAT2, Rao et al. hypothesize that their results remain largely consistent with the premise that BD is characterized by hyperglutamatergic signaling.^142^ Of note, riluzole, which increases the expression of EAAT2, has shown some efficacy in treatment-resistant depression and as adjunctive therapy for bipolar depression in mostly open-label studies.^126, 143^

Another study found decreased expression of the neuronal transporters, EAAT3 and/or EAAT4 but not EAAT1 or EAAT2 in the striatum of MDD and BD (as well as schizophrenia) samples relative to controls.^144^ The decrease in EAAT3 and EAAT4 expression may diminish the capacity of the synapse to clear glutamate, resulting in increased levels of synaptic glutamate in mood disorders.^144^ Supporting the potential relevance of EAAT3 function to depression and psychosis, a deletion of the solute carrier family 1 member 1 (SLC1A1) gene that codes for EAAT3 was found to co-segregate with psychosis (BD or schizophrenia) in a large five-generation pedigree, increasing disease risk by 18-fold.^145^ Similarly, both rare and more common variants of the genes coding for EAAT1 and EAAT2 have been reported to be associated with BD.^146^

Interestingly, autoantibodies to the water channel, aquaporin-4 (AQP4) which are usually associated with neuromyelitis optica, have also been reported in one case of schizophrenia^147^ and a case of treatment-resistant depression.^148^ EAAT2 and AQP4 exist in astrocytic membranes as a macromolecular complex and the binding of AQP4 IgG has been shown to cause the rapid internalization of both AQP4 and EAAT2.^149^

After synaptic uptake into astrocytes by EAATs, glutamate is converted into glutamine by glutamine synthetase and glutamine is, in turn, delivered to neurons where it is re-converted into glutamate. Thus, the expression of glutamine synthetase may affect glutamate cycling and availability to neurons. QA not only inhibits the uptake of glutamate by astrocytes but inhibits astroglial glutamine synthetase^150^ perhaps explaining previous reports of decreased glutamine synthetase expression in the amygdala and PFC of suicide completers.^138, 151^

### Oligodendroglia

**Figure 4:**
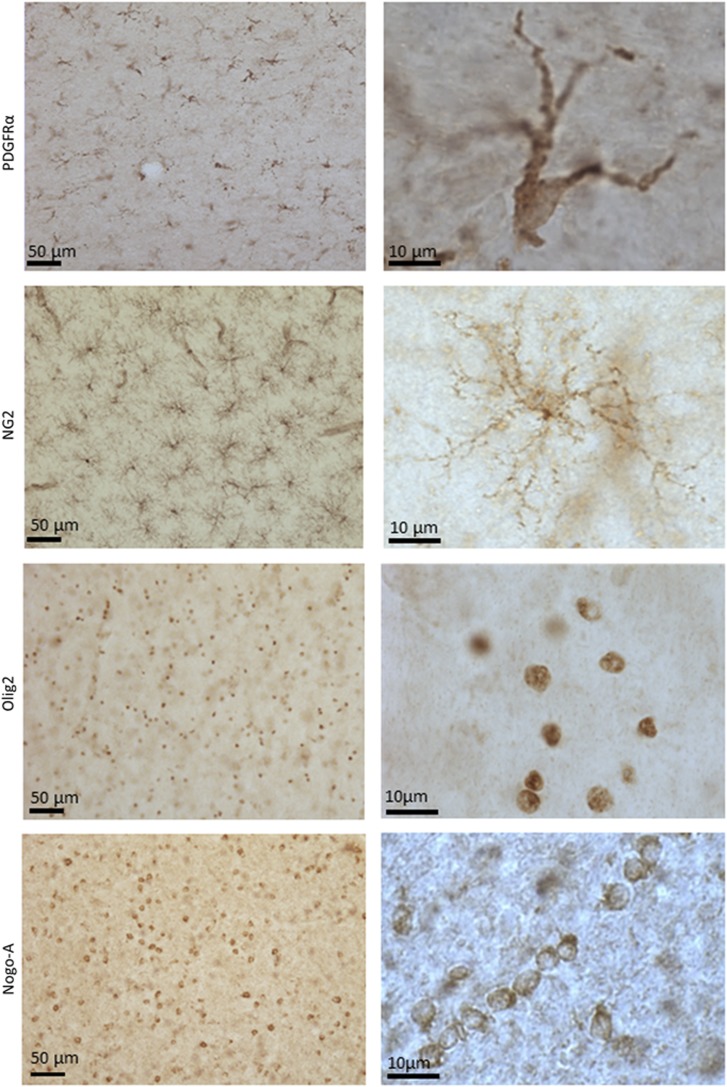
Representative micrographs of immunostained oligodendrocyte precursor cells (PDGFRα- and NG2-immunoreactive), oligodendrocyte-lineage cells (Olig2-immunoreactive) and mature oligodendrocytes (Nogo-A-immunoreactive) in ventromedial prefrontal cortex white matter at low (left column) and high (right column) magnifications. Note the characteristic distribution of Nogo-A+ mature oligodendrocytes along axonal fibers.

Relative to other cell types, oligodendroglia (Figure 4) are particularly vulnerable to inflammation and/or oxidative stress-induced excitotoxicity during development in both rodents and humans.^152, 153^ Lipopolysaccharide is toxic to oligodendrocytes when they are co-cultured with microglia, and in rodents, administration of endotoxin preferentially damages oligodendrocytes; an effect that may be mediated by TLR4 as transgenic mice lacking TLR4, through which endotoxin primarily acts, are less vulnerable to oligodendrocyte damage.^154, 155^ Oligodendroglia in turn are capable of modulating immune function by, for instance, producing CCL-2 (previously known as MCP-1) and IL-1 post injury to open up the blood–brain barrier and recruit peripheral immune cells into the brain parenchyma as well to redirect themselves to areas of demyelination.^156, 157^ Interestingly, inflammation-associated white matter damage is associated with pre-term births, and the risk for BD putatively increases monotonically with (shorter) length of gestation.^158^ Nevertheless, pre-term birth is more generally associated with a variety psychiatric and neurodevelopmental disorders, and thus the correlation between pre-term birth and risk for mood disorders is not necessary indicative of an underlying inflammatory etiology.

Reductions in numbers or density of glia cells—most likely oligodendroglia cells are one of the most prominent findings in postmortem mood disorder samples.^1, 159^ A reduction in the number of glial cells together with an increase in neuronal density initially was found in the subgenual ACC in BD and MDD samples, although the identity of the abnormal glia subtype could not be determined with the nonspecific staining methods applied.^160^ These nonspecific glia cell abnormalities were extended to the supragenual ACC, BA9 of the PFC, the amygdala and the entorhinal cortex. Cotter *et al.* reported a 22% decrease in the glial cell density of layer VI of the supragenual ACC and 30% decrease in the glial cell density in layer V of BA9 in MDD, but not BD, samples.^161, 162^ On the other hand, after a stereological analysis of Nissl-stained tissues, Hercher *et al.*^163^ found no difference in glial densities nor in neuronal densities and average neuronal soma size between supracallosal ACC (BA24a) samples from MDD individuals vs matched controls. However, those samples from MDD subjects who had also been alcohol dependent had significantly higher densities of glial cells in this region compared with the samples from controls or from MDD subjects who were not alcohol dependent.^163^ Further, in the BA9 samples derived from BD subjects, Rajkowska *et al.*^164^ did find evidence for a decrease in the density (16–22%) of glial cells in layer III in conjunction with glial cell enlargement. These discrepancies in the literature may be related to various confounders, including treatment with lithium and valproic acid. Indeed, Price and colleagues reported reductions in glial cell density in the amygdala (and to a lesser extent the entorhinal cortex) of MDD samples but not in the BD samples.^165^ However, further analysis showed that the reduction in glial cell density was in fact present in the untreated BD cases.^165^

Follow-up studies using methods that allowed for the differentiation of oligodendrocytes from other glial cell types, specifically reported reduced numbers of oligodendroglia in layers III and VI of BA9 in BD.^166, 167^ Similarly, a 19% reduction in oligodendrocyte density in the BD samples that reached trend-level significance was found in the amygdala,^168^ while signs of necrosis or apoptosis-related damage to oligodendrocyte cells have been observed in the BD samples from the caudate.^169^ Further, immunoreactivity of myelin basic protein, a surrogate marker of myelination that is expressed by oligodendroglia, was decreased in layer I of the hippocampus in female, but not male subjects with BD.^170^ In the case of MDD, the staining of deep white matter in the dorsolateral PFC was reported to be significantly less intense in MDD subjects compared with controls.^171^ Subsequent studies using specific staining methods or flow activated cell sorting methods, reported reduced numbers of oligodendroglia cells in the amygdala^168^ and frontopolar cortex.^172^

These morphological studies receive support from studies that have measured oligodendrocyte cell-related gene and protein expression changes in mood disorders. For instance, Bahn and colleagues performed a quantitative PCR analysis of BA 9 tissue, finding a significant reduction in mRNA expression of key protein markers of myelination and oligodendrocyte function.^173^ Specifically, the expression of proteolipid protein 1, myelin associated glycoprotein, oligodendrocyte specific protein, myelin oligodendrocyte glycoprotein, and transferrin (TF) was reduced by 2- to 4-fold in the BD patients relative to psychiatrically healthy controls, while the transcription factors, OLIG2 and SOX10, which are involved in oligodendrocyte differentiation and maturation, were downregulated by 2- to 3-fold in BD.^173^ Although this decrease in oligodendrocyte-related gene expression conceivably could result from cell loss, not all of the oligodendrocyte-related genes were downregulated, suggesting that these abnormalities are more likely to reflect cellular dysfunction than cell death.^173^

Because oligodendrocytes are responsible for the myelination of axons, the abnormalities of oligodendroglia observed postmortem appear congruent with the results of diffusion tensor imaging studies, which have produced evidence of structural abnormalities of white matter tracts connecting the prefrontal cortex with limbic nuclei in MDD^174^ and BD.^175, 176, 177^ Interestingly, in patients with BD, a recent study reported a significant positive association between several pro-inflammatory cytokines and radial diffusivity, and an inverse association between these cytokines and fractional anisotropy, leading Benedetti *et al.*^178^ to hypothesize that inflammation is associated with demyelination or dysmyelination in BD. Oligodendrocytes are also involved in the turnover of *N*-acetylaspartate, a marker of neuronal integrity. Males with BD were reported to display a reduced concentration of (*N*-acetylaspartate), in the dorsolateral PFC (BA8, BA9, BA10 and BA46; ref. 179) and similar findings have been published in the pediatric BD literature.^180, 181^ Consistent with these data, *N*-acetylaspartate levels increased significantly in MDD patients after treatment with ECT.^182^

Reductions in numbers or density of oligodendroglia in mood disorder samples, postmortem, also receives support from the preclinical literature. In mice, ablation of nerve/glial antigen 2 (NG2)-expressing oligodendrocytes or a chronic social stress-induced decrease in their density, leads to an impairment of astrocytic glutamate reuptake and depressive-like behaviors which can be rescued by repopulation with endogenous oligodendrocyte progenitor cells.^183^

Given the evidence discussed above for mood disorders, it is tempting to draw parallels with the psychopathological features observed in multiple sclerosis (MS). Indeed, MS is a chronic neuroinflammatory disorder that is characterized by a compromised blood–brain barrier, infiltration of immune cells, and loss of myelin and oligodendrocytes.^184^ Interestingly, MDD co-morbidity is high in MS, with a lifetime prevalence rate of about 50%, that is, 3- to 5-fold that of the general population.^185^ Similarly, BD in MS patients is twice as frequent as in the general population.^185^ Furthermore, MS patients can also present pseudobulbar affect (emotional incontinence), with prevalence estimates varying widely in the literature (6.5–95%).^186^ Pseudobulbar affect, which is often misdiagnosed as MDD or BD, is characterized by inappropriate and uncontrolled crying and/or laughing, and is thought to result from a dishinibition within the corticopontine-cerebellar circuits.^187^ Given these epidemiological and clinical observations, it can be speculated that the altered connectivity arising from neuroinflammation-related oligodendroglial loss and myelination deficits may represent a common path leading to disorders of mood and affect.

### Neurons

#### The NMDA receptor complex

The NMDA receptor is membrane-bound ligand-gated Ca^2+^ channel that is assembled from four or five subunits with an obligatory NR1 subunit associated with different combinations of NR2A–D subunits. The NR1 gene is alternatively spliced, producing eight different isoforms of the NR1 subunit, which combine with different NR2 and NR3 subunits to produce NMDA receptors with distinct pharmacological properties. Activation of the receptor requires multiple signals, that is, glutamate binding, AMPA receptor-mediated depolarization of the postsynaptic membrane, and binding of glycine or d-serine to the NR1 subunit.^188^ The NMDA receptors are found both in the synapse and in extrasynaptic locations on neurons but are predominantly expressed in the postsynaptic membrane where they interact with an intracellular protein complex termed the postsynaptic density (PSD).

Pro-inflammatory cytokines such as IL-1, IL-6 and TNF can enhance the release of glutamate from presynaptic neurons and increase NMDA receptor currents in postsynaptic neurons potentially leading to excitotoxicity.^189^ For instance, IL-1 can enhance NMDA signaling by inducing NR1 (ref. 190) and NR2B (ref. 191) subunit phosphorylation. In neuronal cell cultures, the administration of IFN-α caused dendritic atrophy, an effect that was partially mediated by the NR2A receptor.^192^ Similarly, using whole-cell patch-clamp electrophysiological recordings, Di Filippo *et al.*^193^ reported that IFN-β, a medication that blocks type I interferon receptors and is used to treat infectious and autoimmune diseases such as MS, reduces striatal excitatory postsynaptic potentials through the NR2A NMDA receptor subunit. Further, prenatal inhibition of the kynurenine pathway enzyme, kynurenine monooxygenase, was shown to cause a significant increase in levels of NR2A, NR2B and the postsynaptic NMDA receptor complex protein postsynaptic density protein 95 (PSD-95) at postnatal day 21 together with neuronal excitability and long-term potentiation in the hippocampus.^194^

Regarding postmortem studies, a western blot analysis showed approximately 50% reductions in NR2A, NR2B (but not NR1) as well as the anchoring protein, PSD-95, in the anterior region of the PFC of MDD samples.^195^ In contrast, elevations in NR2A and NR2C and PSD-95 were reported by the same group to be elevated in the locus coeruleus and lateral amygdala, respectively.^196, 197^ A more recent study using quantitative PCR in a large sample of 53 MDD subjects and 32 controls reported increased expression of all the NMDA receptor subunit genes in BA9/BA46 of depressed females, an effect that was accentuated in the individuals who had committed suicide.^198^

In BD, *in situ* hybridization studies generally have reported decreased expression of NR1. Specifically, decreased expression of NR1 was observed in the *Cornu Ammonis 3* (CA3) and hippocampal subiculum of BD samples with a history of psychosis,^199^ while decreased expression of synapse- associated protein 102 (SAP102), NR1 and NR2A (but not NR2B, NR2C and NR2D) was independently reported in hippocampal samples from individuals with BD (but not schizophrenia) relative to controls.^200^ Partially consistent with these data, NR1 (but no other NMDA subunit) expression was decreased in the oriens layer of CA1 but not CA2/3.^201^ Regarding the frontal cortex, NR1 expression was reported to be decreased in the dorsolateral PFC of MDD, BD and schizophrenia samples vs controls.^202^ Similarly, decreased mRNA and protein levels of NR1 and NR3A (but not NR2A or NR2B) together with increased concentrations of inflammatory and excitotoxicity markers such as IL-1, nuclear factor-κB and inducible nitric oxide synthase were reported in BD samples relative to controls.^108^

In aggregate, this literature is suggestive of abnormal glutamatergic signaling in depression. Although the changes in expression of the NMDA receptor subunits could conceivably result from the direct actions of pro-inflammatory cytokines, there may be many other factors driving these changes such as compensatory up- or downregulation of receptors as a result of astrocyte dysfunction-mediated decreases or increases in glutamate levels.

#### Vesicular glutamate transporters

The kynurenine metabolite, xanthurenic acid, which is produced by the transamination of 3HK (Figure 1) and is upregulated in the brain by lipopolysaccharide,^203^ is an endogenous inhibitor of vesicular glutamate transporters (VGLUT).^204^ The vesicular glutamate transporters are responsible for loading glutamate into synaptic vesicles at excitatory neurons thereby regulating presynaptic glutamate release.^205^ VGLUT1 primarily is expressed in the cerebral cortex, hippocampus and cerebellar cortex, whereas VGLUT2 transcripts are expressed subcortically (including in dopamine neurons) and in layer V of the cerebral cortex.^206, 207^ VGLUT3 is expressed in the subsets of serotonergic, cholinergic and GABAergic interneurons.^206^

Eastwood and Harrison reported increased VGLUT1 expression in the supragenual ACC (BA24) in BD, a result they hypothesize is indicative of abnormally increased glutamate neurotransmission, rather than, or in addition to, alterations in glutamate metabolism or cycling.^208^ Consistent with these data, Gray *et al.*^198^ reported increased VGLUT1 expression in the dorsolateral PFC of female MDD samples using quantitative PCR. This abnormality may be region-specific since decreased expression of VGLUT1 also has been reported in mood disorders. Using *in situ* hybridization, Uezato *et al.*^206^ reported decreased VGLUT1 mRNA expression in both MDD and BD in the entorhinal cortex as well as decreased VGLUT2 expression in MDD, (trending for BD) in the middle temporal gyrus. Consistent with these data, decreased expression of VGLUT1 was reported in layer V of both MDD and BD samples from the dorsolateral PFC (BA9).^209^ A reduction in VGLUT1/2 mRNA may be due to structural loss of presynaptic terminals or alternatively a functional decrease in glutamate release while conversely, increased VGLUT expression may be indicative of greater numbers of glutamate neurons and/or greater presynaptic innervation and glutamate neurotransmission.^198, 206^

#### GABAergic interneurons

Structural and/or functional changes to GABAergic interneurons are one of the most robust postmortem findings in BD.^1^ For instance, in the rostral ACC, Woo *et al.*^210^ found a reduction in the density of NR2A-expressing GABAergic interneurons in subjects with BD (and schizophrenia) relative to controls and in a separate study, the density of GABAergic neurons expressing the GluR5 subunit of the kainate receptor was found to be decreased by 40% in layer II of this region.^211^ Similarly, the density of calbindin (CB)-expressing GABAergic neurons in layer II of the supracallosal gyrus was reduced by 33% in BD^212^ and in the dorsolateral cortex, the density of glutamic acid decarboxylase 67 (GAD_67_) mRNA-containing (GABAergic) neurons in layers II through V of BA9 was decreased by approximately 25–33%.^213^ These data are supported by additional studies reporting decreased expression of GABAergic neuron-associated proteins such as GAD_67_ in both cortical and subcortical regions,^214, 215, 216, 217^ as well as a meta-analysis showing a reduction in the density of CB-expressing neurons in layer VlI of BA9 in BD.^218^ Similarly, MDD appears to be characterized by reduced density of GABAergic interneurons labeled with calretinin and/or CB in the dorsolateral PFC,^219^ occipital cortex^220^ and auditory cortex.^221^

These data suggest that GABAergic neurotransmission in at least a subset of local neuronal circuits is attenuated; however, the functional implications are not well understood. In addition to disinhibition of pyramidal neurons, other studies demonstrate that parvalbumin-containing interneurons are critical for the generation of gamma oscillations,^222, 223^ which are implicated in normal cortical function (for example, working memory^224^). Expression of mRNA for neuronal activity-regulated pentraxin, a protein that is secreted at presynaptic glutamate synapses that terminate on parvalbumin-containing interneurons, is reduced in the dorsolateral PFC of subjects with BD,^225^ suggesting that excitatory drive onto this interneuron subclass is disrupted which, in turn, could lead to a disruption of gamma oscillations and associated cortical function.

The mechanisms through which inflammation specifically affects the structure and/or function of GABAergic interneurons is not yet clear. However, there are preclinical data showing that inflammation impacts GABAergic circuits. Multiple rodent studies have reported that prenatal exposure to lipopolysaccharide or polyinosinic: polycytidylic acid (poly I:C) is associated with a reduction in the density of parvalbumin and/or GAD_67-_staining neurons in the hippocampus (reviewed in ref. 226). Potentially consistent with these data, Clements *et al.*^227^ found a significant reduction in parvalbumin-staining interneurons in layer II of the motor cortex in MS patients vs controls. Stress may have a modulating role in the relationship between inflammation and GABAergic function. For instance, maternal separation stress in rats causes a reduction of GABAergic parvalbumin-expressing interneurons in the PFC; an effect that was reported to be blocked by intracerebroventricular administration of the anti-inflammatory cytokine, IL-10.^228^ Similarly, adult offspring born to viral mimetic poly(I:C)-exposed mothers who were subjected to unpredictable subchronic stress during peripubertal development displayed significant reductions of parvalbumin-expressing interneurons in the dentate gyrus.^229^

In a different vein, *T. gondii* infection was shown to shift the distribution of GAD_67_ expression such that GAD_67_ becomes diffusely located throughout the neuropil rather than clustering on presynaptic termini where it catalyzes GABA synthesis in the brain.^230^

## Caveats

### Cause or effect?

Although we have emphasized the potentially detrimental effects of neuroinflammation on the brain, microglial cells also exert neuroprotective effects. Toll-like receptor signaling caused by cellular damage or infectious agents triggers a rapid activation of microglial cells, clearing away debris, promoting angiogenesis, neurogenesis, increasing the recruitment of oligodendrocyte progenitor cells, favoring remyelination and conferring neuroprotection.^231^ However, without the rapid clearance of myelin debris or toxins from the brain, microglial cells may instead mount a detrimental pro-inflammatory response, resulting in demyelination, synaptic dysfunction and ultimately neurodegeneration.^231, 232, 233^ Thus a delicate equilibrium between under- and overactivation of microglial cells may determine whether the conditions conducive to neuronal repair or neuronal damage, predominate. There are likely to be many mechanisms beyond removal of debris and oligodendroglia-genesis underlying these neuroprotective effects. For instance, activated macrophages and microglial cells express high-affinity glutamate transporters and glutamine synthetase during both acute and chronic central nervous system inflammation, potentially partially compensating for astrocytic dysfunction.^234^ Mice given intraperitoneal injections of 1.0 mg kg^−1^ of endotoxin for four consecutive days to globally activate central nervous system microglia, are protected from a subsequent traumatic brain injury challenge^235^ by microglia-mediated stripping of inhibitory GABAergic synapses, which facilitates NMDA receptor signaling allowing for greater expression of neurotrophic and anti-apoptotic molecules such as brain-derived neurotrophic factor and B-cell lymphoma 2 (Bcl-2).^236^ Thus conceivably, the increased expression of glutamate-related genes together with the decreased expression of GABA observed in postmortem tissue in BD may be an adaptive response to injury rather than a cause of neuronal pathology.

### Misattribution of function

The assumption that molecules that are involved in the peripheral immune response automatically play the same functional role in the brain is potentially faulty. A case in point is the recent discovery that a variant in the gene coding for a component of the complement (CA4) pathway constitutes a risk factor for schizophrenia.^237^ Rather than (or at least in addition to) defending against microbial infection, CA4 in the brain appears to have a key role in synaptic pruning during neurodevelopment.^237^ Thus postmortem alterations in inflammation-related proteins do not necessarily reflect an immune-related etiology—at least in the classical sense of the word.

## Conclusion and future directions

The most prominent morphological abnormalities observed in postmortem samples from individuals with BD include decreases in the density and/or number of GABAergic neurons and glial cells. The literature describing microglial activation in mood disorders is smaller but appears robust. These data are complemented by gene expression studies, which are consistent with functional deficits in myelination, astrocytic glutamate recycling and in the regulation of inhibitory/excitatory neurotransmission. Immune processes could conceivably account for these abnormalities, but with the possible exception of the microglia data it is not possible to draw a direct link between neuroinflammation and histopathology. It is likely that there is a great deal of heterogeneity within the MDD and BD syndromes with respect to pathophysiology and etiology such that postmortem samples encompass patients with a range of conditions that appear clinically related but are neurobiologically distinct. This lack of a precise and biologically verifiable definition of illness, together with contrasting experimental approaches presumably contribute to the inconsistencies observed within the literature. Future studies should give greater consideration to these factors to better clarify the possible contribution of neuroinflammation to the etiology of mood disorders.

It will be particularly importance for future postmortem studies to be carried out using thoroughly characterized samples, that is, case and control tissues for which substantial clinical information is available and complemented retrospectively with psychosocial data. Moreover, in addition to common tissue-based immunohistochemical and molecular approaches, the field should increasingly take advantage of techniques allowing for single cell analyses, such as laser capture microdissection and fluorescence-activated cell sorting. These approaches offer the opportunity to explore and compare, within a given brain region or circuit, the epigenetic and molecular profiles of different cellular subtypes (neurons vs glia, subtypes of neurons and glia, macrophages and so on). Such characterizations should lead to a more detailed understanding of the causes and consequences of cerebral neuroinflammation in mood disorders.
